# Six-Month In Vivo Safety Profiling of Topical Ocular AVV5–Decorin Gene Transfer

**DOI:** 10.1167/tvst.10.10.5

**Published:** 2021-08-12

**Authors:** Rajiv R. Mohan, Praveen K. Balne, Maryam S. Muayad, Ratnakar Tripathi, Nishant R. Sinha, Suneel Gupta, Jella A. An, Prashant R. Sinha, Nathan P. Hesemann

**Affiliations:** 1 Harry S. Truman Memorial Veterans’ Hospital, Columbia, Missouri, USA; 2 One-Health Vision Research Program, Departments of Veterinary Medicine & Surgery and Biomedical Sciences, College of Veterinary Medicine, University of Missouri, Columbia, MO, USA; 3 Mason Eye Institute, School of Medicine, University of Missouri, Columbia, Missouri, USA

**Keywords:** cornea, AAV5-decorin gene therapy, tolerability, safety, decorin

## Abstract

**Purpose:**

A significant remission of corneal fibrosis and neovascularization in rabbit eye in vivo was observed from a tissue-selective localized adeno-associated virus (AAV)5–*Decorin* (*Dcn*) gene therapy. This study sought to investigate 6-month toxicity profiling of this gene therapy for the eye in vivo using a rabbit model.

**Methods:**

A small epithelial scrape followed by corneal drying was performed unilaterally in 12 rabbit eyes and either AAV5–*Dcn* (*n* = 6) or naked vector (*n* = 6) was delivered topically using a cloning cylinder technique. Contralateral eyes served as naïve control (*n* = 6). Safety and tolerability measurements in live rabbits were performed periodically until month 6 using multimodel clinical ophthalmic imaging tools—a slit lamp, stereomicroscope, and HRT3-RCM in vivo confocal microscope. Thereafter, corneas were excised and subjected to hematoxylin and eosin staining, Mason trichome staining, propidium iodide nuclear staining, and quantitative real-time polymerase chain reaction analyses.

**Results:**

Clinical eye examinations based on the modified Hackett–McDonald ocular scoring system, and in vivo confocal imaging of the cornea showed no signs of ocular toxicity in rabbit eyes given AAV5–*Dcn* gene transfer vs control eyes (*P* > 0.05) through 6 months after treatment. The histologic and molecular analyses showed no significant differences in AAV5–*Dcn* vs AAV naked or naïve control groups (*P* > 0.05) and were in accordance with the masked clinical ophthalmic observations showing no abnormalities.

**Conclusions:**

Topical tissue-targeted localized AAV5–*Dcn* gene therapy seems to be safe and nontoxic to the rabbit eye in vivo*.*

**Translational Relevance:**

AAV5–*Dcn* gene therapy has the potential to treat corneal fibrosis and neovascularization in vivo safely without significant ocular toxicity.

## Introduction

Gene therapy is an emerging modality to treat inherited and acquired corneal diseases. The cornea is an ideal tissue for gene therapy approaches owing to its easy accessibility for delivering agents, quick and noninvasive examinations, and immune-privileged status.^1^^–^^3^ The recent studies in corneal gene therapy led to the development of various treatment modalities for various corneal disorders including inherited dystrophies, allograft rejection, herpetic stromal keratitis, corneal haze/fibrosis, opacity, and neovascularization.^4^^–^^14^ One of the challenges in advancing corneal gene therapy approaches from bench to bedside is the lack of comprehensive long-term safety profiling, because any later adverse effect can impair vision. The efficacy of several genes for treating corneal defects in vivo in preclinical animal models has been reported, but studies on the long-term tolerability and safety are still lacking. For gene therapy to become a clinical reality, it is imperative to examine the long-term tolerability of successful genes and the carrier vectors.

Adeno-associated virus (AAV) has emerged as one of the favorite vectors for gene therapy applications in the cornea. AAV was discovered as contaminants of adenovirus stocks and hence called an adeno-associated virus.^1^^–^^3^ AAV vectors are nonenveloped, single-stranded DNA viruses belonging to the genus *Dependovirus* of the *Parvoviridae* family. *Dependoviruses* require a helper virus to infect and cannot replicate on their own making them safe for gene therapy. The recombinant AAV used in gene therapy lacks viral DNA and contains the DNA of therapeutic genes to be delivered. Once recombinant AAV enters the cells, its DNA exists nucleus as an AAV episome, which does not integrate with the host genome, and as cells divide it reduces over time. This decrease of episomal DNA in the cells leads to the loss of delivered therapeutic gene expression. The rate of loss of the delivered gene depends on the turnover rate of the cells that receive the gene of interest. These characteristics make AAV vectors attractive and safe for gene therapy application, especially for medical conditions that do not demand constant high sustained gene expression in cells.^3^ Specific serotypes of AAV interact with specific carbohydrates and co-receptors on the cell surfaces and such interaction confers cell-tropism. AAV5 uses sialic acid to enter the corneal stromal keratocytes.^1^^,^^2^ AAV vectors are considered superior for delivering genes to corneal keratocytes.^1^^,^^15^^–^^18^ A simple minimally invasive customized vector delivery technique using corneal epithelial scrape followed by air drying the corneal surface and delivering the gene via custom-made cloning cylinder was developed to overcome the corneal epithelial barrier and deliver therapeutic levels of genes topically into corneal stromal keratocytes.^19^

AAV5 vectors have been used to deliver the decorin gene in the rabbit cornea.^8^^,^^9^ Decorin, a small leucine-rich proteoglycan, is endogenously expressed in the stroma and regulates the size of collagen fibrils.^20^ Furthermore, it binds and regulates transforming growth factor-β activity, which is an important mediator of fibrotic response in the injured cornea.^21^ Decorin is a potent antiscarring molecule shown to have significant antifibrotic effects in glaucoma and corneal wound healing. Intracameral injection of human recombinant decorin significantly decreased transforming growth factor-β–induced trabecular meshwork fibrosis, intraocular pressure (IOP), and prevented retinal ganglion cell loss in a rat model trabecular meshwork fibrosis.^22^ Chouhan et al.^23^ developed a microstructured fluid gel eye drop system to deliver decorin into the wounded cornea and reported that the fluid gel containing decorin enhanced corneal re-epithelialization in ex vivo organ culture model and in vivo rat model of corneal wound healing. Our earlier studies found localized tissue-targeted AAV5–*Dcn* gene therapy given topically in the stroma highly efficacious in treating corneal haze or fibrosis^8^ and neovascularization^9^ in vivo in rabbit experimental models with unremarkable acute adverse effects. In the used rabbit in vivo disease models, corneal fibrosis was produced by common refractive laser surgery, photorefractive keratectomy.^8^ Corneal neovascularization was induced by implanting a vascular endothelial growth factor pellet in a corneal micropocket.^9^ Although these studies uncovered the therapeutic promise of AAV5–*Dcn* gene therapy for treating corneal pathology and vision loss, they did not provide any information about its long-term safety. This study examined the 6-month-long tolerability and safety of AAV5–*Dcn* gene therapy for the eye in vivo using a rabbit model using clinical eye examinations, multimodel ophthalmic imaging tools, and histologic and molecular biological techniques.

## Methods

### Animals

The study was approved by the Institutional Animal Care and Use Committee of the University of Missouri, Columbia, Missouri, and all the animals used in this study were treated as per the ARVO Statement for the Use of Animals in Ophthalmic and Vision Research. Twelve New Zealand White rabbits (Charles River Laboratory Inc., Wilmington, MA) with weight ranging from 2.2 to 3.0 kg were used in this study. Rabbits were divided into three groups (naïve, naked vector, and AAV5–*Dcn* gene delivered). Rabbit's unilateral eyes were topically administered AAV5–*Dcn* gene therapy (*n* = 6), or naked vector (*n* = 6), and the contralateral eyes (*n* = 6) were used as naïve controls. Rabbits were housed in individual cages in environmentally controlled rooms at the University of Missouri's animal facility, and they were fed certified feed and water ad libitum.

### Generation and Delivery of AAV5–*Dcn*

The *Dcn* expressing recombinant AAV5 titer was generated and delivered into the rabbit cornea following our previously reported protocols.^8^^,^^9^ Briefly, an intramuscular injection of a mixture of ketamine hydrochloride (50 mg/kg; JHP Pharmaceuticals, LLC, Rochester, MI) and xylazine hydrochloride (10 mg/kg; XylaMed, Bimeda Inc., IL) was given to rabbits for general anesthesia, and a drop of topical ophthalmic 0.5% proparacaine hydrochloride (Alcon, Fort Worth, TX) was given for local anesthesia. The corneal epithelium was removed with number 64 Beaver blade (Becton–Dickinson, Franklin Lakes, NJ) by gentle scraping under an operating microscope. The eyes were washed with a copious amount of balanced salt solution (BSS) (Alcon, Geneva, Switzerland) and wiped with a merocel sponge after removing the corneal epithelium. The rabbit corneas were then dried with a hair-dryer, operated from an approximate distance of 8 inches and 45° angle to the eye, blowing warm air thrice for 10 seconds with 5-second interval as reported elsewhere.^19^ Thereafter, a 50 µL titer of AAV5–*Dcn* (6.5 × 10^12^ µg /mL, *n* = 6) or AAV5 naked (6.5 × 10^12^ µg/mL, *n* = 6) vector was topically applied for 2 minutes using a cloning cylinder. The remaining vector was soaked in a merocel sponge and eyes were washed with BSS.

### Clinical Evaluations and Multimodel Ophthalmic Imaging

The clinical ocular evaluations and multimodel ophthalmic imaging in live rabbits before and after AAV5 gene delivery, at day 3, and 1, 3, and 6 months were performed under general and local anesthesia with a slit lamp microscope, stereomicroscope, confocal microscope, fluorescein test, pachymetry, tonometry, and Schirmer tear test. Topical BSS was used to prevent the rabbit eyes from desiccation during the process of clinical examinations and ophthalmic imaging.

A portable slit lamp microscope (Kowa, SL-15, Torrance, CA) coupled with a high-definition digital imaging system (Kowa, portable VK-2 Ver. 5.50) was used for ophthalmic clinical examination. Rabbit corneas were also imaged with a stereo-microscope (Leica MZ16F, Leica Microsystems Inc., Buffalo Grove, IL) equipped with a digital camera (SpotCam RT KE, Diagnostic Instruments Inc., Sterling Heights, MI). A fluorescein stain (Altafluor Benox, Sigma Pharmaceuticals, North Liberty, IA) test was used to assess corneal epithelial defects. Changes in the IOP were measured by a Tono-Pen (AVIA Tonometer, Scottsdale, AZ), and central corneal thickness (CCT) was measured with an Ultrasonic pachymeter (Accutome, AccuPach VI Pachymeter, Malvern, PA). Schirmer tear test strips (Fisher Scientific, Pittsburgh, PA) were used to measure changes in tear flow. A modified Hackett–Mcdonald scoring system was used to evaluate the safety and tolerability of AAV5–*Dcn* gene therapy to the rabbit eyes and was performed by at least two independent examiners.^24^ The corneal cellular corneal architecture at different layers was studied using HRT3-RCM in vivo confocal microscopy (Heidelberg Engineering, GmBH, Dossenheim, Germany) and 400  ×  400 µm pictures of the superficial and intermediate epithelium, the corneal stroma, and the endothelium were taken in the center of the cornea.

### Corneal Tissue Collection

Rabbits were humanely euthanized at 6 months with an intravenous injection of pentobarbital (150 mg/kg; Diamondback Drugs, Scottsdale, AZ) while the animals were under general anesthesia. After humane euthanasia, the cornea was dissected and cut into two halves: one-half cornea (*n* = 6/group) was immediately placed into 24 × 24 × 5-mm mold (Fisher Scientific, Pittsburgh, PA) containing optical cutting temperature compound (Sakura Finite, Torrance, CA), snap frozen in liquid nitrogen by immersing in 2-methyl butane (Sigma-Aldrich), and stored at −80 °C until sectioning. The remaining half cornea (*n* = 6/group) was cut into another half and directly placed into cryovials, immersed in liquid nitrogen, and used for RNA (*n* = 6/group), DNA (*n* = 3/group), and protein (*n* = 3/group) extractions. Eight-micron thick corneal sections were prepared using Microm HM525 cryostat (Thermo Fisher Scientific, Waltham, MA), mounted on microscopic glass slides (Superfrost Plus, Fisher Scientific, Pittsburgh, PA), and preserved at −80 °C for histologic evaluation.

### Histologic and Immunofluorescence Evaluations

To study the effect of gene delivery on corneal anatomy, rabbit corneal tissue sections from the naïve and AAV5-naked, and AAV5–*Dcn* groups were subjected to hematoxylin and eosin (H&E) and Mason trichome staining following our published protocols.^25^^,^^26^ The H&E and Mason trichome stained corneal sections were imaged with a bright-field microscope (Leica DM 4000B, Leica Microsystems Inc.) equipped with a digital camera and imaging software (SpotCamRT KE; Diagnostic Instruments, Sterling Heights, MI). The effect of gene therapy on cellular density was studied by mounting corneal sections with an antifade Vectashield mounting medium with propidium iodide (PI) (Vector Laboratories, Inc., CA). The PI-stained nuclei in corneal sections were recorded with a fluorescence microscope (Leica DM 4000B, Leica Microsystems Inc.) equipped with a digital camera (SpotCam RT KE, Diagnostic Instruments Inc.). The cellular density in the corneas of the naïve, AAV5 naked, and AAV5–*Dcn* groups was quantified by counting PI-stained nuclei^27^ in ten randomly selected areas in corneal sections at 200 and/or 400 magnification field following the method reported by our laboratory earlier.^21^

### Molecular Evaluations

The effect of AAV5–*Dcn* gene therapy at the molecular level was evaluated through quantitative real-time polymerase chain reaction (qRT-PCR) and enzyme-linked immunosorbent assay (ELISA) at 6 months after therapy. Corneal tissues were minced in a tissue lyser (TissueLyser LT, Qiagen, Valencia, CA) and total RNA and DNA were extracted using a commercial RNA extraction (RNeasy Mini Kit, Qiagen) and DNA extraction kits (QIAamp DNA Mini Kit, Qiagen) following the manufacturer's instructions. The cDNA was synthesized using Avian Myeloblastosis Virus Reverse Transcriptase (Promega, Madison, WI) following the manufacturer's guidelines^25^ and used for alpha-smooth muscle actin (*α-SMA*) gene expression analysis and DNA was used for gene copy number analysis. A 20 µL qRT-PCR reaction mixture containing 1 µL of cDNA or DNA, 3 µL of 200 nM forward primer, 3 µL of 200 nM reverse primer, 10 µL of 2X All-in-One PowerUp SYBR green master mix (Applied Biosystems, Carlsbad, CA), and 3 µL of RNAse/DNAse free water; was run at the universal thermal cycling conditions (95 °C for 10 minutes, 40 cycles at 95 °C for 15 seconds, and 60 °C for 60 seconds) in StepOnePlus Real-Time PCR system (Applied Biosystems). *Beta-actin* (*β-actin*) was used as a housekeeping gene and primer sequences *α-SMA* forward 5′-TTGACTGAGGCACCGCTGAA-3′ and reverse 5′-CCACGTACATGGCTGGGACA-3′ and *β-actin* forward 5′- TTGGAGCGAGCATCCCCAAA -3′ and reverse 5′- GGCTTCCGTCACATGGCATC-3′ were used to study the corresponding genes messenger RNA expression. The relative messenger RNA expression of the genes was calculated using the 2^−ΔΔCt^ method and reported as relative fold change.^28^ For gene copy analysis serial dilutions of pTRUF11-*Dcn* plasmid from 10^9^ to 10^1^ copies/µL were used to generate a standard curve and copy number analysis was done for AAV5–*Dcn* gene delivered rabbit corneas using primer sequences UF11-internal-forward 5′- TTGGCGAATTCGAACACGCAGATG-3′ and UF11-internal-reverse 5′- ATGGATACTTTCTCGGCAGGAGCA-3′, by absolute quantification method. Protein was extracted from homogenized rabbit corneal tissues using RIPA protein lysis buffer containing protease inhibitor cocktail (Roche Applied Sciences, Indianapolis, IN) and DCN protein concentration was measured using rabbit DCN ELISA kit using manufacturer's guidelines (LSBio).

### Statistical Analysis

Statistical analysis was performed using GraphPad Prism 9 software (GraphPad Software, La Jolla, CA). One-way analysis of variance (ANOVA) with the Tukey post-hoc test was used to determine statistical significance for IOP, CCT, tear flow, modified Hackett-Mcdonald scores, PI-stained nuclei, qRT-PCR, and ELISA data. The *p* < 0.05 was considered statistically significant.

## Results

### Clinical Ophthalmic Examinations and Multimodel Imaging

**Figure 1.**
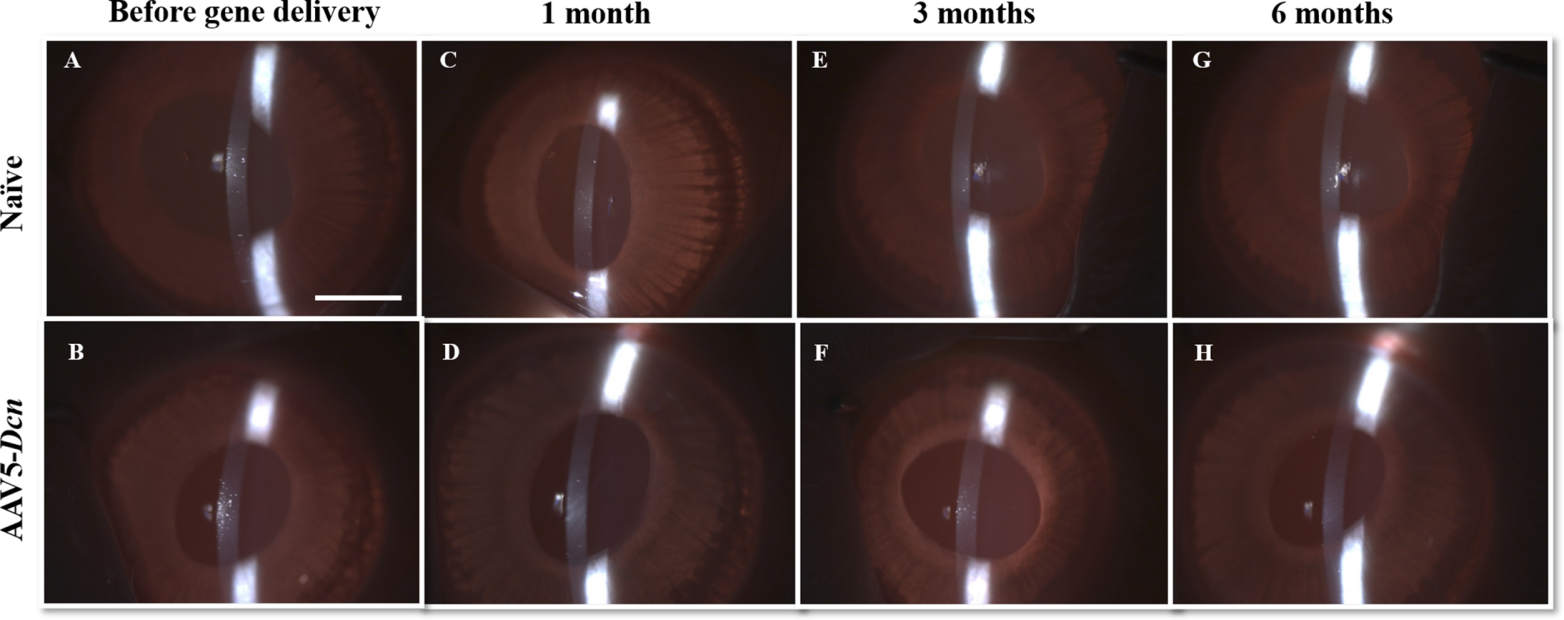
Representative in vivo slit-lamp images revealing the optical sections of the cornea in the naïve (*n* = 6) and AAV5–*Dcn* (*n* = 6) delivered rabbit eyes. (A and B), (C and D), (E and F), and (G and H) represent the images of before gene delivery, and after 1 month, 3 months, and 6 months of gene delivery, respectively. AAV5–*Dcn* gene therapy caused no significant toxicity to the cornea and showed corneal health similar to naïve (*P* > 0.05). Naked vector–treated rabbit eyes (*n* = 6) showed images similar to AAV5–*Dcn* (not shown). Scale bar = 2.0 mm.

**Figure 2.**
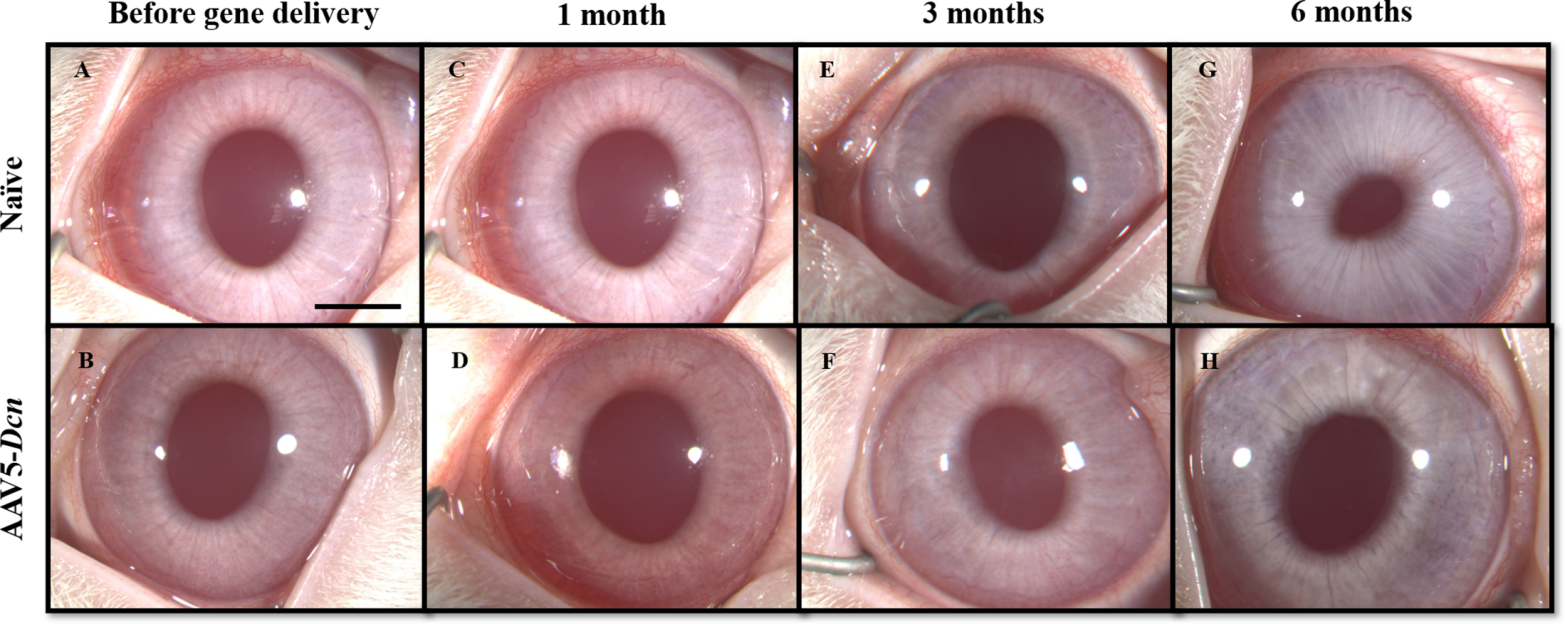
Representative in vivo stereomicroscopic images revealing the corneal health in the naïve (*n* = 6) and AAV5–*Dcn* delivered (*n* = 6) rabbit eyes. (A and B), (C and D), (E and F), and (G and H) represent the images of before gene delivery, after 1 month, 3 months, and 6 months of gene delivery, respectively. AAV5–*Dcn* gene therapy caused no long-term toxicity to the cornea (*P* > 0.05). Naked vector-treated rabbit eyes (*n* = 6) showed images similar to AAV5–*Dcn* (not shown). Scale bar = 2.0 mm.

Slit-lamp ophthalmic examination and optical sections of the cornea in the AAV5–*Dcn* groups appeared normal until the longest tested time, six months. No cloudiness or defects in corneal epithelium, stroma, or endothelium was observed (Fig. 1). The slit lamp (Fig. 1) and stereo biomicroscopy examinations (Fig. 2) revealed no conjunctival congestion for six months in the AAV5–*Dcn* gene or -naked vector delivered eyes. The conjunctiva appeared clear, while conjunctival and perilimbal vessels remained normal, specifically without signs of inflammation. No swelling in the bulbar conjunctiva or palpebral conjunctival on eversion of the eyelids was noted until six months. Also, no serous, purulent, mucoid, or bloody discharge was seen from the conjunctiva until six months. No corneal vascularization (pannus) was observed in the AAV5–*Dcn* gene delivered eyes for six months. No hyperemia was observed in the iris vessels of the AAV5–*Dcn* delivered rabbit eyes.

### Fluorescein Staining

**Figure 3.**
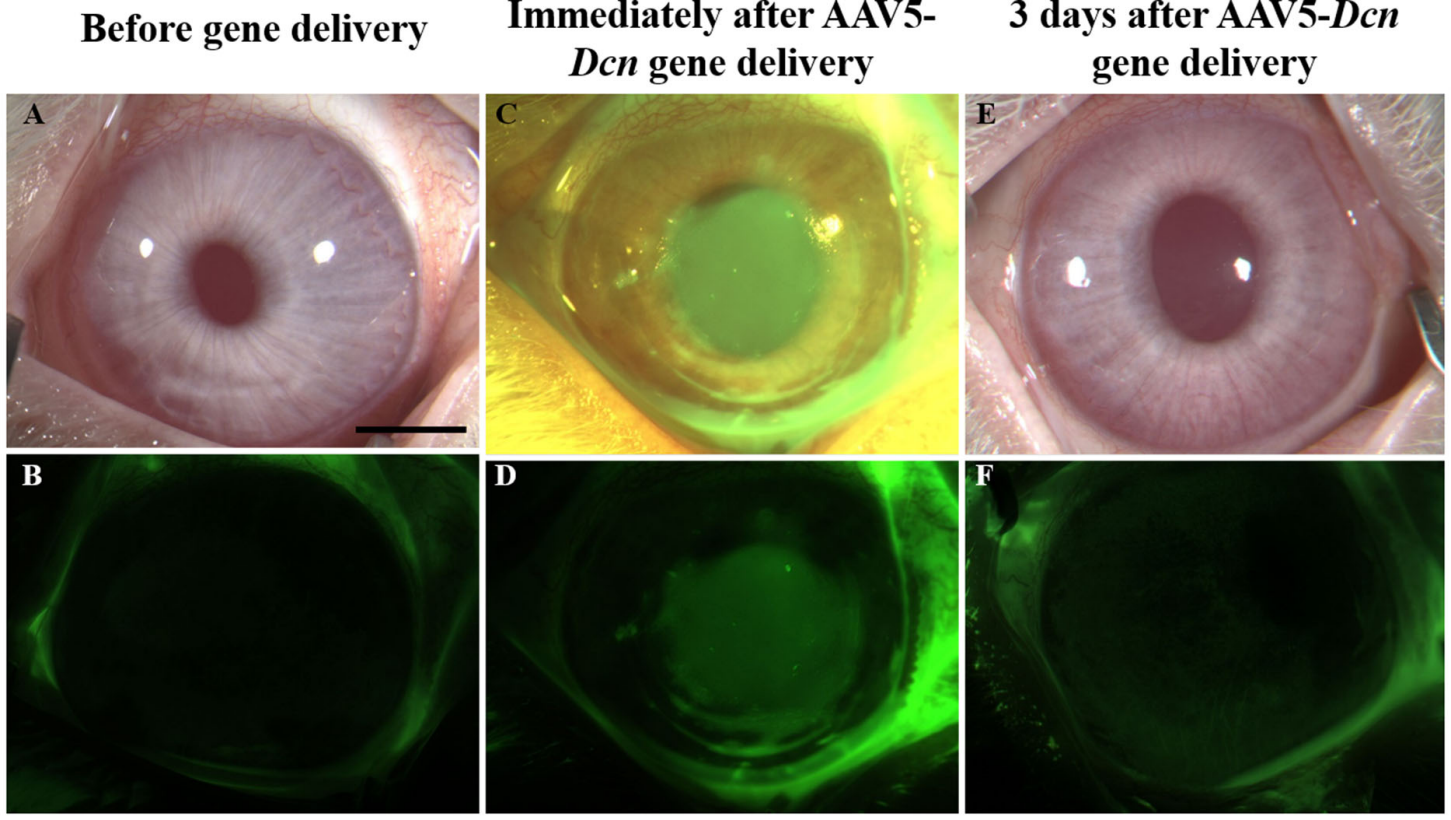
Initial ocular evaluations in AAV5–*Dcn* delivered rabbit corneas. (A and B), (C and D), and (E and F) show the representative in vivo rabbit eye images revealing ocular health and fluorescein uptake before gene delivery, immediately after gene delivery, and at 3 days after gene delivery, respectively. Rabbit corneas were found healthy throughout the study duration except for an expected fluorescein uptake in the AAV5–*Dcn* therapy group immediately after gene delivery was noted due to epithelial removal to facilitate gene therapy in the stroma (C and D), which healed fully by day 3 (E and F). Scale bar = 2.0 mm.

**Figure 4.**
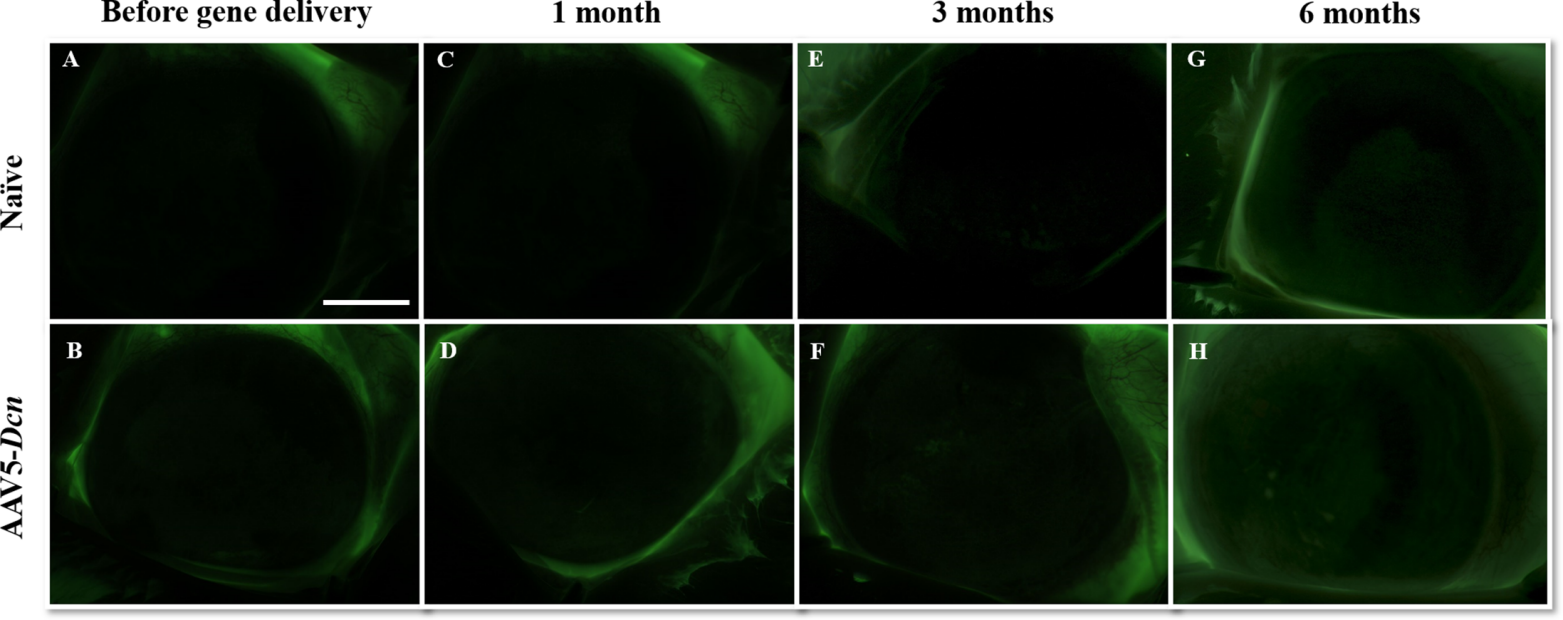
Representative in vivo stereomicroscopic images under green fluorescence filter after application of fluorescein dye revealing no fluorescein uptake in the naïve (*n* = 6) and AAV5–*Dcn* delivered (*n* = 6) rabbit eyes. (A and B), (C and D), (E and F), and (G and H) represent the images before gene delivery, after 1 month, 3 months, and 6 months of gene delivery, respectively. Naked vector-treated rabbit eyes (*n* = 6) showed images similar to AAV5–*Dcn* (not shown). No significant differences among corneas of the three groups were noted throughout the study (*P* > 0.05). Scale bar = 2.0 mm.

Fluorescein staining determined the damage to the corneal epithelium (Fig. 3). The bright-field images are shown in Figs. 3A, C, and E, and fluorescein images are shown in Figs. 3B, D, and F. Before gene therapy, rabbit corneas were normal and showed no fluorescein uptake (Figs. 3A and B). Conversely, an expected fluorescein uptake was observed immediately after AAV5–*Dcn* therapy because epithelium was removed to facilitate gene therapy in the stroma (Figs. 3C and D). Corneal epithelium healed completely by day-3 after gene therapy as no fluorescein uptake was detected in the AAV5–*Dcn* delivered cornea (Figs. 3E and F). The health of regenerated corneal epithelium in the AAV5–*Dcn* delivered eyes was compared with the corneal epithelium of naïve rabbit eyes (Fig. 4). The corneas of AAV5–*Dcn* delivered eyes were normal throughout the testing period as no fluorescein uptake was noticed at 1, 3, or 6 months (Fig. 4).

### Modified Hackett–McDonald Ocular Scoring System

The modified Hackett–Mcdonald ocular scoring system recorded the observations of slit-lamp examinations for evaluating the toxicology profile in rabbits. The Modified Hackett-Mcdonald scores showed no abnormal changes under the headings of pupillary light reflex, conjunctival congestion, conjunctival swelling, conjunctival discharge, corneal opacity, and percentage area of corneal opacity, corneal neovascularization, iris congestion, fluorescein staining, and percentage area of fluorescein staining.^24^ No abnormal scores were obtained in any of the categories of the Modified Hackett-Mcdonald test in the AAV5–*Dcn* delivered eyes.

### IOP

Measurement of IOP provides information about the dynamics of aqueous flow in the eye. The means of IOP in AAV5–*Dcn*, naked vector, and naïve rabbit eyes before gene delivery were 7.83 ± 0.91, 9.67 ± 0.67, and 9.33 ± 0.62, respectively and was gradually increased to 10.33 ± 0.34, 10.33 ± 0.84 and 10 ± 0.58, respectively, at the end of six months. The difference in IOP was statistically insignificant (*P* > 0.05) for three groups at the six months. Table shows the changes in IOP noted in rabbit eyes at different time points. An expected slight increase in IOP was observed in rabbit eyes because of the age-related changes.^29^

**Table.** Tear Flow, CCT, and IOP Measurements in Rabbit Eyes at Different Time Points

		IOP		CCT		Tear Flow	
Timepoints	Groups	Mean	SEM	Mean	SEM	Mean	SEM
Before gene delivery	Naïve	9.33	0.615	353.67	5.315	11.83	0.703
	Naked vector	9.67	0.667	347.17	5.231	12.50	1.118
	AAV5–decorin	7.83	0.910	346.00	9.220	13.67	1.606
After gene delivery	Naïve	9.83	1.222	350.83	3.798	12.50	1.455
	Naked vector	12.50	1.565	442.00	21.650	**24.83** ^*^	2.120
	AAV5–decorin	9.67	1.174	407.17	36.895	**25.83** ^*^	2.286
Day 3	Naïve	9.50	0.563	353.17	4.902	12.17	1.424
	Naked vector	9.50	0.885	382.67	8.110	14.83	1.922
	AAV5–decorin	7.67	1.085	360.17	9.799	13.00	2.236
1 month	Naïve	10.17	1.078	349.67	3.138	10.50	1.088
	Naked vector	9.67	0.760	353.17	3.728	11.33	1.382
	AAV5–decorin	9.50	0.563	350.83	6.503	15.83	2.574
3 months	Naïve	9.83	1.249	353.50	5.045	12.33	0.667
	Naked vector	9.50	0.764	358.17	3.250	10.67	0.989
	AAV5–decorin	10.50	0.764	356.33	7.419	15.83	2.330
6 months	Naïve	10.00	0.577	353.33	4.240	11.67	1.085
	Naked vector	10.33	0.843	352.67	4.485	12.50	1.803
	AAV5–decorin	10.33	0.333	369.67	9.006	15.50	0.85

* *P* < 0.05 for comparisons with naïve group.

SEM = standard error of the mean.

### CCT

CCT was measured in all three groups to assess corneal edema. The mean CCT in AAV5–*Dcn*, naked vector and naïve rabbit eyes before gene delivery were 346.00 ± 9.22 µm, 347.18 ± 5.23 µm, and 353.67 ± 5.32 µm, respectively. At the terminal 6-month time, the CCT for AAV5–*Dcn*, naked vector, and naïve rabbit eyes were found 369.67 ± 9.01 µm, 352.67 ± 4.49 µm, and 354.33 ± 4.24 µm, respectively. The CCT measurements had no significant differences among the three groups (*P* > 0.05) throughout the study (Table).

### Schirmer Tear Test

A Schirmer tear test detected basal and reflex tear production and aided to assess dryness in the rabbit eye. Before gene delivery, the mean tear flow values in the AAV5–*Dcn*, naked vector, and naïve rabbit eyes were 13.67 ± 1.61 mm, 12.5 ± 1.12 mm, and 11.83 ± 0.70 mm, respectively. An expected transient increase in tear flow was observed in AAV5–*Dcn* and naked vector groups (mean ± standard error of the mean 25.83 ± 2.29 and 24.83 ± 2.12, respectively; *p* = 0.05). The increase in tears was due to the mild trauma to corneal epithelium caused by the vector-delivery technique. However, no significant changes in IOP and CCT were noted after gene or naked vector introduction (*P* > 0.05) in the cornea by the employed topical vector-delivery technique (Table). The tear flow values were back to normal in AAV5–*Dcn* and naked vector groups (13 ± 2.24 and 14.83 ± 1.92, *p* = 0.05) within 3 days, and thereafter no significant variations were noted until 6 months (Table) between the AAV5–*Dcn* and naked vector eyes (15.5 ± 0.85 mm and 12.5 ± 1.8 mm, *P* > 0.05). The naïve rabbit eyes showed no significant change in tear flow until 6 months (mean ± SD 11.67 ± 1.1 mm, *P* > 0.05). The tear measurements had no significant variations among the three groups at the end of six months (*P* > 0.05).

### In Vivo Confocal Microscopy

**Figure 5.**
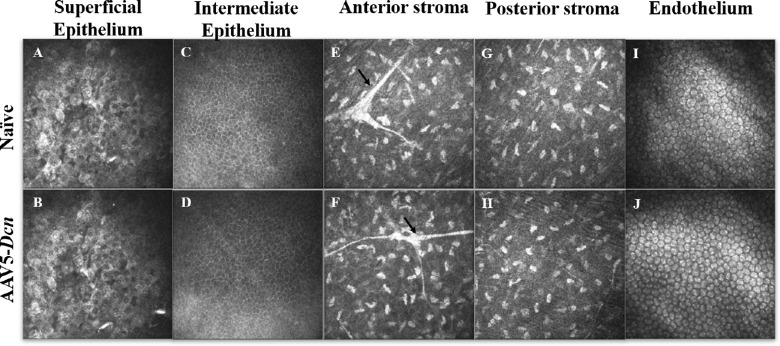
Representative in vivo confocal microscopic images obtained with the HRT3-RCM of different layers of the live rabbit cornea in the naïve (*n* = 6) and AAV5–*Dcn* delivered (*n* = 6) eyes at 6 months. Images of the superficial epithelium (A and B), intermediate epithelium (C and D), anterior stroma (E and F), posterior stroma (G and H), and endothelium (I and J) of AAV5–*Dcn* delivered rabbit corneas demonstrated no significant differences (*P* > 0.05) with the naïve corneas. The arrows in the anterior stroma depict the nerve fibers. All images are 400 × 400 µm.

The treated and untreated rabbit eyes were also subjected to in vivo confocal microscopy using an HRT3-RCM biomicroscopy system in a real-time manner (1-month, 3-month, and 6-month) to study the corneal architecture at the level of the epithelium, stroma, and endothelium. The in vivo confocal images collected at various times from the corneas of three groups (AAV5–*Dcn,* naked vector, and naïve) appeared similar and exhibited no clinical abnormalities until the tested 6-month period. Figure 5 shows in vivo clinical confocal imaging data procured at 6-month (1-month and 3-month data were similar and therefore not shown).

The in vivo confocal images of superficial corneal epithelium of AAV5–*Dcn* delivered eyes (Fig. 5B) appeared similar to the naïve rabbit corneas (Fig. 5A) at six months. Superficial epithelial cells were large, polygonal, had bright cytoplasm with reflecting nucleus, and perinuclear halo in both the groups (AAV5–*Dcn* and naïve), which is typical of this cell type. Also, there was no desquamation, irregular cell shapes, cellular swelling, cell border loss, inflammatory infiltration, and abnormal reflectivity patterns observed in the superficial epithelium of both groups (Fig. 5). The superficial epithelium of the naked vector delivered corneas was similar to the naïve corneas too (data not shown). Likewise, the intermediate epithelial cells of the AAV5–*Dcn* delivered corneas (Fig. 5D) looked similar to naïve rabbit corneas (Fig. 5C) at six months. Intermediate epithelial cells (or wing cells) formed a regular mosaic with sharp and reflecting cellular borders in both the groups (AAV5–*Dcn* delivered and naïve). No disorganization or inflammatory infiltration was observed in the intermediate epithelium of both groups. The intermediate epithelium of naked vector treated rabbit corneas showed morphologic features similar to AAV5–*Dcn* delivered rabbit corneas (data not shown).

The in vivo confocal images of the anterior stroma of the AAV5–*Dcn* delivered corneas (Fig. 5F) seemed similar to naïve rabbit corneas (Fig. 5E) at 6 months. The anterior stroma had keratocyte nuclei, which appeared as reflecting light corpuscles, and the connecting lamella, which appeared black in both the groups (AAV5–*Dcn* delivered and naïve). The sub-basal nerve plexus in the anterior stroma of both groups was observed as hyper-reflective fibers. No disorganization or inflammatory infiltration was observed in the anterior stroma of either group. The anterior stroma of naked vector group rabbit corneas showed morphological features similar to AAV5- *Dcn* gene delivered rabbit corneas (data not shown). The in vivo clinical imaging deeper in the cornea reviewing the status of posterior stroma found no differences in appearance, population, and density of keratocytes in AAV5–*Dcn* (Fig. 5H) and naïve corneas (Fig. 5G) until longest tested 6 months. The posterior cornea displayed bright-reflecting keratocyte nuclei in both AAV5–*Dcn* and naïve rabbit eyes (Figs. 5H and G). The posterior stroma of naked vector corneas was similar to the naïve rabbit corneas (data not shown).

The in vivo confocal analysis of the endothelium of AAV5–*Dcn* delivered corneas (Fig. 5J) seemed to be similar to those of the naïve rabbit corneas (Fig. 5I) at 6 months. The treated and naïve eyes of all rabbits showed a classical monolayer of hexagonal-shaped endothelial cells that appeared similar in size, shape, and number (*P* > 0.05). There were no signs of polymegatism and pleomorphism in the endothelium of treated versus nontreated (naïve) eyes.

### Histologic Evaluations

**Figure 6.**
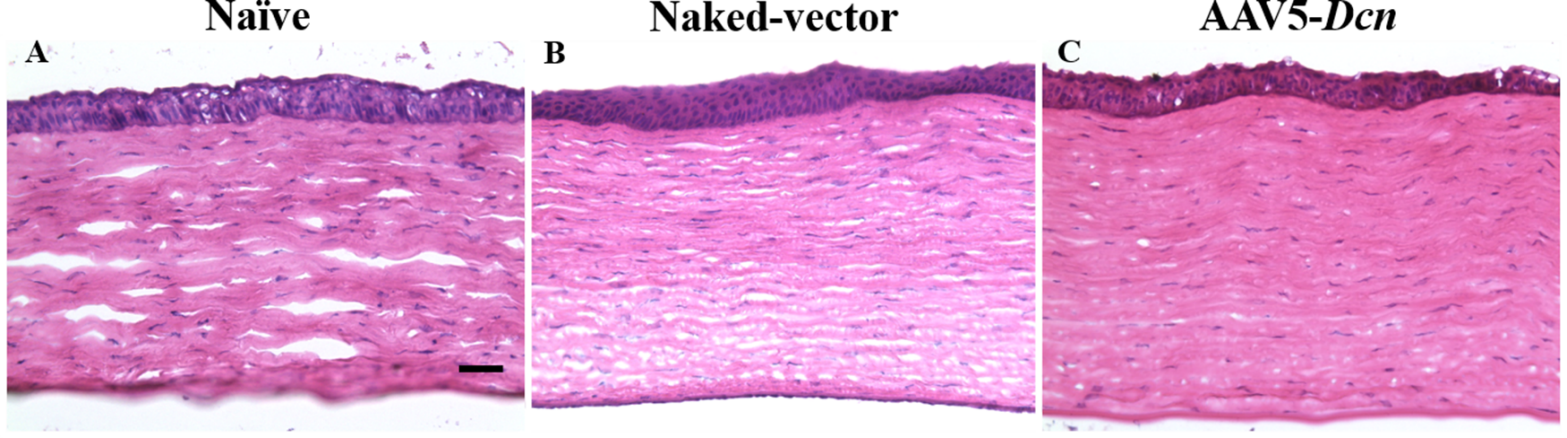
Representative H&E–stained images of corneal tissue sections of naïve (*n* = 6) (A), naked vector (*n* = 6) (B), and AAV5–*Dcn* delivered (*n* = 6) groups (C). No significant variations (*P* > 0.05) in general anatomical layout and distribution of cells within the cornea were observed in all three groups. Scale bar = 200 µm.

We performed the H&E staining on corneal sections of the naïve, naked vector, and AAV5–*Dcn* groups. The stained corneal sections of three experimental groups demonstrated no significant dissimilarities in general anatomical layout and distribution of cells within the cornea (Fig. 6). The H&E stained corneal sections of three groups had three well-defined regions of corneal epithelium, stroma, and endothelium with no significant presence of inflammatory cells (Fig. 6).

**Figure 7.**
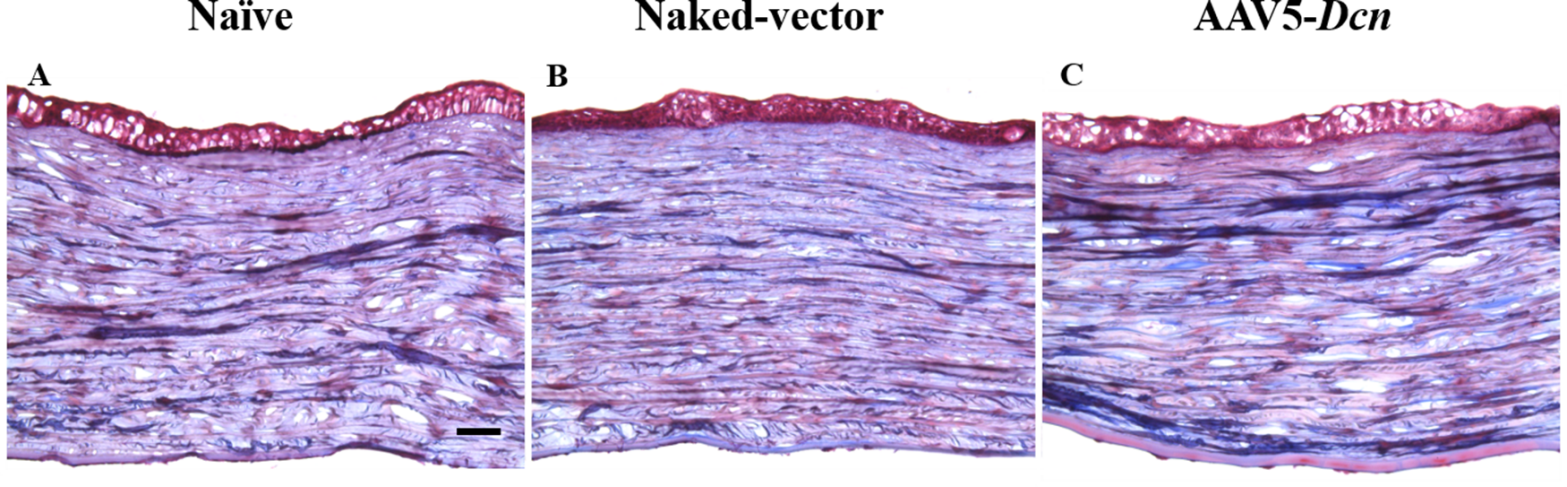
Representative Mason trichome stained images showing qualitatively comparable collagen staining patterns in corneal tissue sections of naïve (*n* = 6) (A), naked vector (*n* = 6) (B), and AAV5–*Dcn* delivered (*n* = 6) groups (C). Scale bar = 200 µm.

Mason Trichome staining was also performed to investigate the status of collagen and signs of abnormal wound healing pattern among the three groups (Fig. 7). This qualitative histologic staining showed unremarkable findings in the corneas of three experimental groups (Fig. 7).

**Figure 8.**
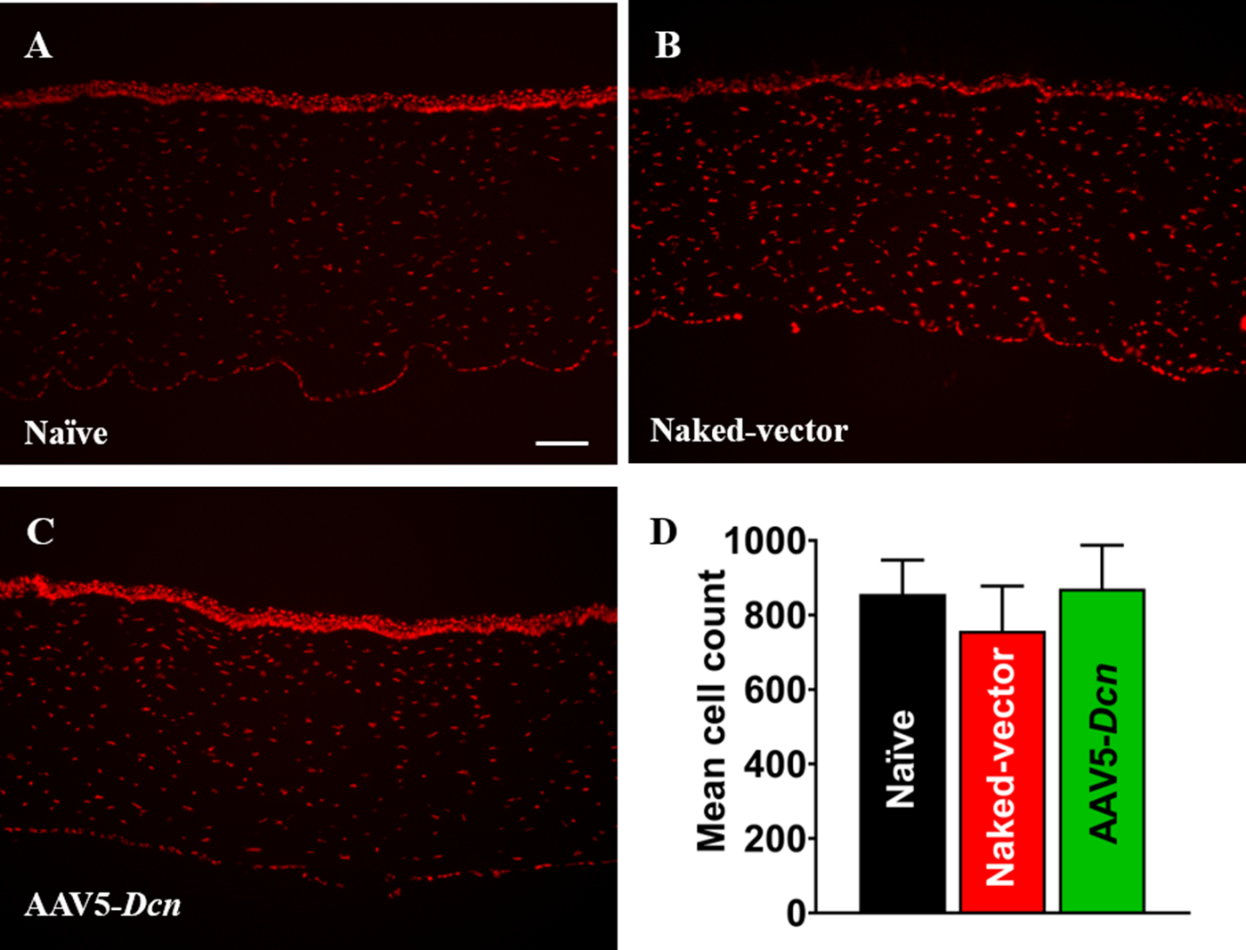
Representative immunofluorescence images of PI-stained nuclei in the naïve (*n* = 6) (A), naked vector (*n* = 6) (B), and AAV5–*Dcn* delivered rabbit corneas (*n* = 6) (C). The population of PI-stained nuclei in naïve, naked vector, and AAV5–*Dcn* delivered corneas were similar and no significant differences among the three groups were noted (*P* > 0.05). Scale bar = 100 µm.

Cellular density is a measure of the epithelial stratification, loss in keratocytes, and increase in the number of infiltrating cells.^30^^,^^31^ The effect of AAV5–*Dcn* gene therapy on the corneal cellular density was evaluated by nuclear staining with PI. The means of cell count in the naïve, naked vector and AAV5–*Dcn* delivered corneal tissues were 856.5 ± 36.1, 757.72 ± 34.8, and 871.11 ± 40.3, respectively (Figs. 8A–C). The quantification analysis found no statistically significant (*P* > 0.05) differences among the corneas of the three experimental groups (Fig. 8D). There were also no noticeable differences in the epithelial stratification between the groups.

### Molecular Evaluations

**Figure 9.**
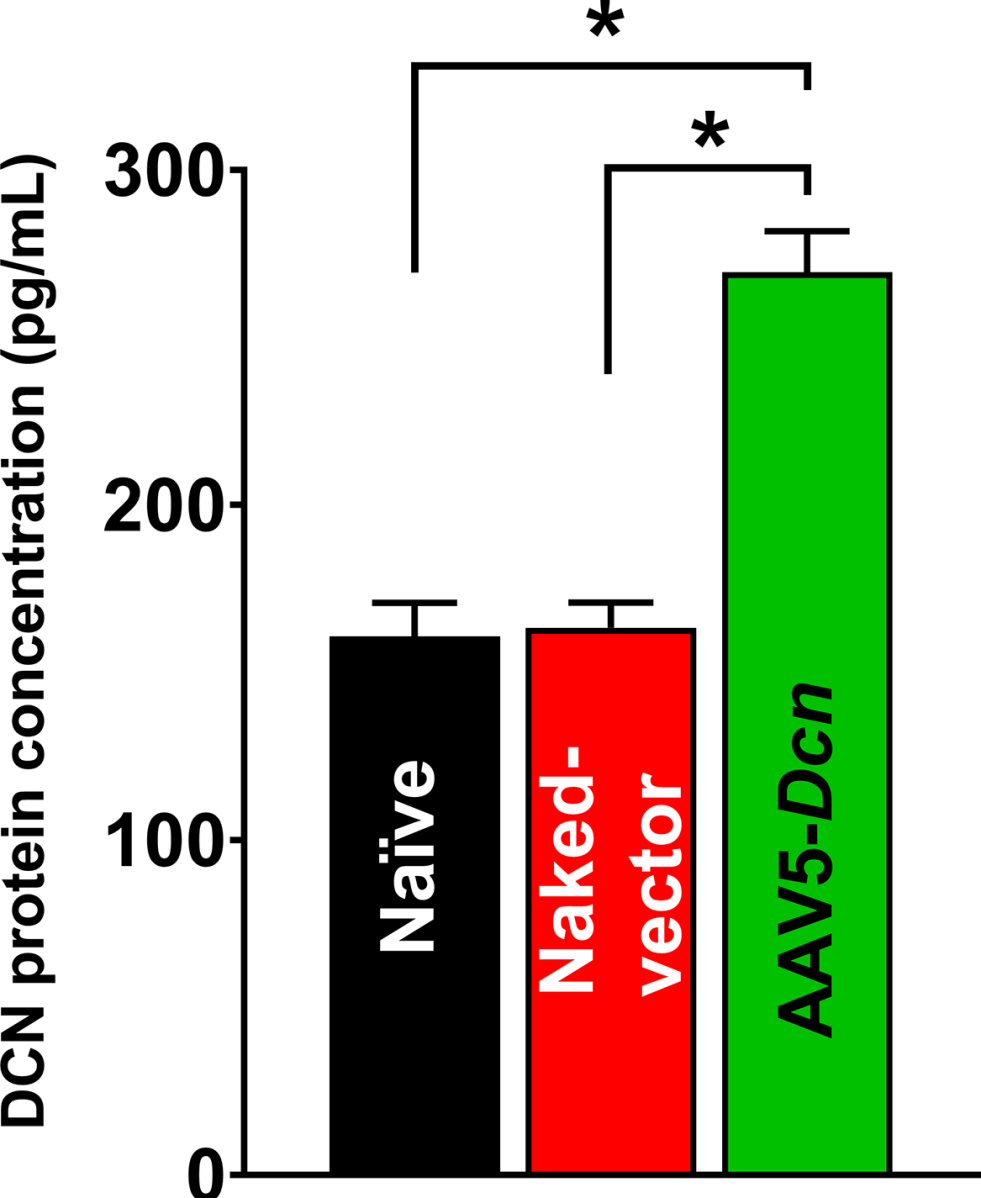
Rabbit decorin (DCN) ELISA results showing DCN protein expression in the naïve, naked vector, and AAV5–*Dcn* delivered rabbit corneas at the end of six months. AAV5–*Dcn* delivered rabbit corneas showed significant upregulation of DCN protein expression compared with naïve and naked vector groups (*n* = 3; *P < 0.05).

**Figure 10.**
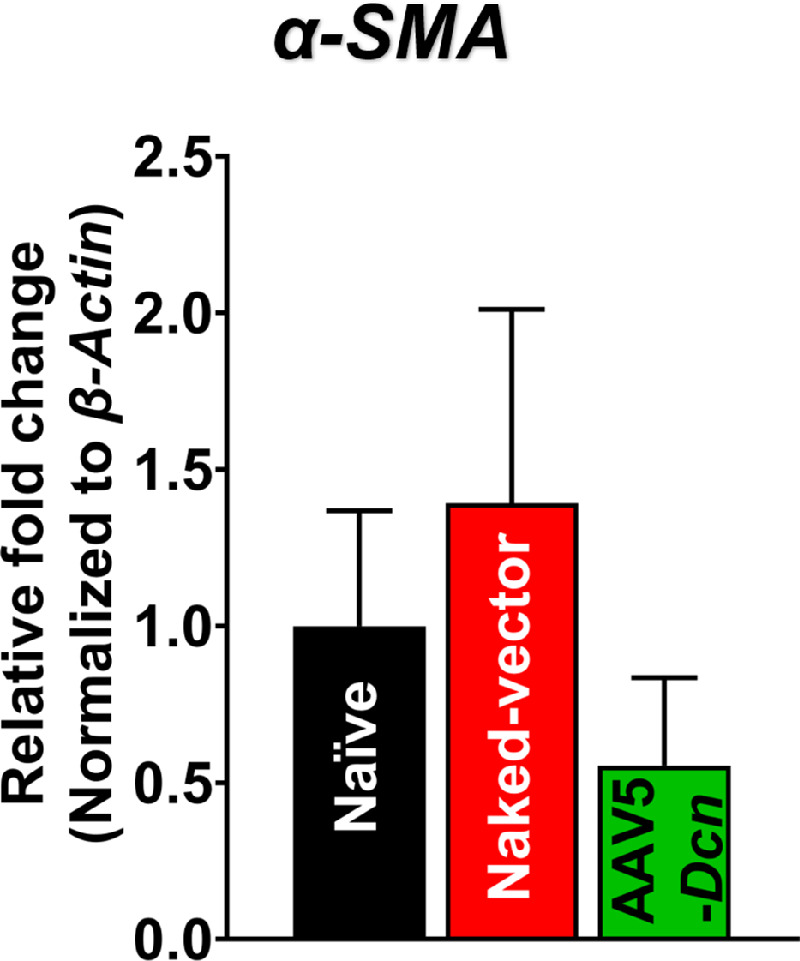
A qRT-PCR analysis showing relative mean fold change in *α-SMA* gene expression in the naïve, naked vector, and AAV5–*Dcn* delivered rabbit corneas at the end of 6 months. AAV5–*Dcn* delivered rabbit corneas showed no significant differences in *α-SMA* gene messenger RNA expression compared to naïve and naked vector groups (*n* = 6; *P* > 0.05).

ELISA and qRT-PCR were used to study the effects of AAV5–*Dcn* gene delivery at the molecular level. The average gene copy number of AAV5–*Dcn* in gene delivered rabbit corneas at 6 months after gene delivery was found to be 10^4^ copies. DCN ELISA results showed significantly higher expression of DCN protein in tissue lysates of AAV5–*Dcn* gene delivered rabbit corneas, compared to naïve and naked vector delivered corneas (*P* < 0.05) (Fig. 9). These results validate the AAV5–*Dcn* gene delivery into the rabbit corneal stroma and demonstrate AAV5-mediated *Dcn* transgene and protein expression in the rabbit corneal stroma at 6 months after gene delivery. The expression of *α-SMA* gene messenger RNA was determined in the corneas of the three experimental groups (Fig. 10). *α-SMA* is a component of stress fibers found in the myofibroblasts and is considered a marker of estimating fibrotic response in the cornea. The expression of *α-SMA* in the AAV5–*Dcn* delivered corneas was found to be similar to the expression of *α-SMA* in the naïve and naked vector group corneas (*P* > 0.05) (Fig. 10). This finding suggests that single topical AAV5–*Dcn* gene therapy did not activate the fibrotic response in the cornea.

## Discussion

Gene therapy is an attractive modality for curing corneal diseases and disorders. It has the potential to produce desirable quantities of biologically relevant proteins for a desired period in the cornea. The data on the dose and duration of therapy helps to maneuver the safety and efficacy of a therapy. Safety and tolerability are essential for the bench to bedside translation of corneal gene therapy for human application. Our 6-month-long comprehensive safety analyses applying clinical, histologic, and molecular parameters of AAV5–*Dcn* gene therapy in rabbit eyes found similar to the naïve rabbit eye suggested that topical AAV5–*Dcn* gene therapy is nontoxic and safe to the eyes in vivo*.*

The tissue-targeted delivery of therapeutic genes into corneal stroma via topical application requires vectors to transcend the protective epithelial barrier to access keratocytes in vivo. A variety of simple, minimally invasive vector delivery techniques were developed to introduce therapeutic genes topically into stromal keratocytes in vivo.^1^^,^^19^ Gentle removal of epithelium on a small corneal area followed by seizing of corneal hydration led to significantly increased entry of vectors into the stroma and allowed large clinically desired amounts of therapeutic gene expression in the corneal stroma.^19^ This technique was used while delivering the AAV5–*Dcn* gene in rabbit eyes as evident from the fluorescein uptake noted right after gene transfer (Fig. 3). Consistent with our earlier studies, rabbit epithelium re-epithelizes fully without any defects within 3 days.^32^^–^^34^ The normal turnover of corneal epithelium in humans is about 1 week.^35^ The scraping of the epithelium for gene delivery is minimally invasive as epithelium heals itself within the turnover rate (Fig. 3). Also, removal of the epithelium to facilitate enhanced gene delivery into the stroma did not cause any epithelial defects in the long term as evident from the fluorescein test results at 6 months (Figs. 3 and 4). The Schirmer tear test and pachymetry studies found that the gentle removal of epithelium caused a slight statistically insignificant increase in the tear flow and CCT for a short duration that normalize within 3 days after the initial insult. Scraped corneas are reported to have an increase in corneal thickness, which normalizes by 3 days.^32^ Scraping of the epithelium may irritate the corneal nerves which may result in reflex tear secretion. The initial changes in tear flow, CCT, and IOP were not observed at subsequent follow-ups up to 6 months. which suggested additionally that these changes were not because of the AAV5–*Dcn* gene therapy and were because of the initial epithelial scraping. Other clinical features scored on the modified McDonald–Hackett scale were apparently normal in the AAV5–*Dcn* gene delivered corneas. Furthermore, cellular organization of primary cell types in rabbit corneas in live animals was examined with advanced ophthalmic in vivo confocal microscopy, HRT3-RCM imaging system. The qualitative and quantitative clinical eye imaging did not find any unusual morphologic variations in the corneal superficial epithelium, intermediate epithelium, anterior stroma, posterior stroma, and endothelium of AAV5–*Dcn* delivered rabbit corneas and measurements were analogous to control naïve rabbit corneas (Fig. 5).

AAV vectors can induce cellular and humoral immune responses in the eye.^36^ This was studied by probing the presence of inflammatory cells with histologic H&E and PI nuclear staining. The H&E staining did not reveal any remarkable morphologic anomalies in corneal tissue sections of the AAV5–*Dcn*, AAV naked, and naive groups (Fig. 6). Likewise, PI nuclear staining exhibited no significant differences in the PI-stained nuclei counts in corneas of the AAV5–*Dcn*, AAV naked, and naive groups (Fig. 8). These analyses demonstrated that neither the AAV5 vector nor decorin gene causes an immunogenic response in rabbit cornea in vivo and is safe for delivering gene therapy in the eye. Decorin is known to regulate the organization of collagen in the corneal stroma.^20^ It is reasonable to presume that AAV5–*Dcn* gene therapy may compromise collagen fibrillogenesis in the cornea. To rule out this possibility, we examined collagen levels in the corneas of the experimental and naive groups of rabbits collected 6 months after the inception of gene therapy. The Mason trichome staining performed in corneal tissue sections of naïve, AAV5–*Dcn,* and AAV naked vector delivered rabbit eyes demonstrated no significant differences in collagen staining pattern (Fig. 7). These data indicated that AAV5–*Dcn* gene therapy is safe to ocular tissue. In our earlier report, at 4 weeks after gene delivery, 10^6^ genomic copies of *Dcn* was detected in the AAV5–*Dcn* gene delivered rabbit cornea.^8^ However, in this study at 6 months after gene delivery, the gene copy number was decreased to 10^4^ copies. This observation is consistent with the fact that the time duration of gene expression by AAV vectors is considered as weeks to months.^1^ Corneal epithelial wound not involving the stroma heals without scarring (Fig. 3E and F).^32^^–^^34^ However, these superficial corneal epithelial wounds can induce apoptosis in corneal stromal keratocytes in the anterior stroma. Then the keratocytes located periphery to the injury migrate into the anterior stromal region and replicate.^37^ Recombinant AAV DNA does not integrate with the host genome, and as cells divide it decreases over time. This reasoning could explain the decrease in gene copy number in rabbit corneas at 6 months after gene delivery or it may also be due to rabbit-specific phenomena and needs to be determined in future studies. Although the gene copy number was decreased at 6 months after gene delivery, the expression of DCN protein in AAV5–*Dcn* delivered rabbit corneas was still significantly higher than in naïve and naked vector delivered corneas at 6 months (Fig. 9).

Because the delivery of AAV5–*Dcn* gene into stroma involved epithelial scrape in a small area, we measured the levels of *α-SMA*, a fibrotic marker, to verify if mild trauma to corneal epithelium causes any issue. A detection of *α-SMA* RNA levels in naive and AAV5–*Dcn* delivered corneas (Fig. 10) indicated that mild trauma to the cornea does not compromise corneal refractive power or cause the fibrotic response. Clinically, epithelium scraping is routinely used to treat recurrent epithelial erosions in the cornea in human patients.^38^^–^^41^

Although our study found the clinical, histologic, and molecular parameters in AAV5–*Dcn* delivered eyes similar to the naïve eyes in the long term, there are limitations to our study. The cellular response during normal and traumatic conditions varies greatly in the cornea. The severity of injury and competence of wound repair mechanisms after injury essentially determine the fate of corneal transparency and refractory power.^1^^,^^42^^–^^45^ Stepp et al.^46^ describe the influence of different injury intensities, wounding techniques, animal models, mouse strain, gene alterations (global and tissue-specific), and so on on corneal wound healing. In this study, we chose to evaluate the consequences of the AAV5–*Dcn* gene delivered onto the epithelium scrapped rabbit cornea in vivo. Whether observed long-term safety and tolerability parameters apply to the damaged cornea/eye remains unknown, which can be a limitation of the study. Additionally, only one concentration of AAV5–*Dcn* titer that was found efficacious in treating corneal fibrosis and neovascularization in rabbits in vivo^8^^,^^9^ was used in the study, which can be seen as another weakness of the study. Further time-dependent experiments are also needed to determine the lasting period of effective *Dcn* transgene expression in postgene delivery rabbit corneas. Accumulation of mutant *Dcn* in the cornea is reported to induce stress responses, resulting in bilateral opacities.^47^ Our study did not detect any opacity in the cornea, suggesting that the overexpression of normal *Dcn* gene in stroma does not induce such stress responses. Nevertheless, it is imperative to execute a large comprehensive study for advancing topical simple and effective AAV5–*Dcn* gene therapy from the research setting to human application.

In conclusion, the 6-month follow-up study with AAV5–*Dcn* gene delivery in rabbits reveals that AAV5–*Dcn* gene therapy seems to be safe and tolerable to the eye in vivo. The findings of the present study in conjunction with our earlier efficacy evaluations illustrate that AAV5–*Dcn* gene therapy has a high potential for the treatment of corneal fibrosis and neovascularization in vivo*.*
